# Biotic/Abiotic Stress-Driven Alzheimer's Disease

**DOI:** 10.3389/fncel.2016.00269

**Published:** 2016-11-23

**Authors:** Chang-Qing Li, Qing Zheng, Qi Wang, Qing-Ping Zeng

**Affiliations:** ^1^Tropical Medicine Institute, Guangzhou University of Chinese MedicineGuangzhou, China; ^2^Department of Biopharmaceutics, College of Pharmacy, Jinan UniversityGuangzhou, China; ^3^Clinical Pharmacology Institute, Guangzhou University of Chinese MedicineGuangzhou, China

**Keywords:** Alzheimer's disease (AD), biotic stress, abiotic stress, gut microbiota, opportunistic infection, lipopolysaccharide (LPS), reactive oxygen species (ROS), reactive nitrogen species (RNS)

## Introduction

Alzheimer's disease (AD), a neurodegenerative condition, is characterized by deficient synaptic plasticity, dramatic neuronal dysfunction, and massive neuronal loss. Apart from familial or early-onset AD (5–10%), most AD cases are non-familial or late-onset/sporadic (90–95%; Ballard et al., 2011) with a complicated etiology. Some competing theories have been suggested regarding the cause of AD, such as the amyloid hypothesis (Hardy and Allsop, 1991) and tau hypothesis (Mudher and Lovestone, 2002), but minimal data on initial triggers are available despite intensive explorations over recent decades.

We summarized the published evidence into an opinion that deciphers how the multifaceted adverse environmental factors drive the onset and development of AD. Etiological drivers can be categorized as biotic stressors and abiotic stressors, with the latter category divided into physical stressors and chemical stressors. Ultimately, biotic/abiotic stressors can be integrated into reactive oxygen species (ROS)/oxidative stressors and reactive nitrogen species (RNS)/nitrosative stressors that impact the transition of neurons from dysfunction to death (Barone et al., 2011a,b; Butterfield et al., 2014).

**Figure 1:**
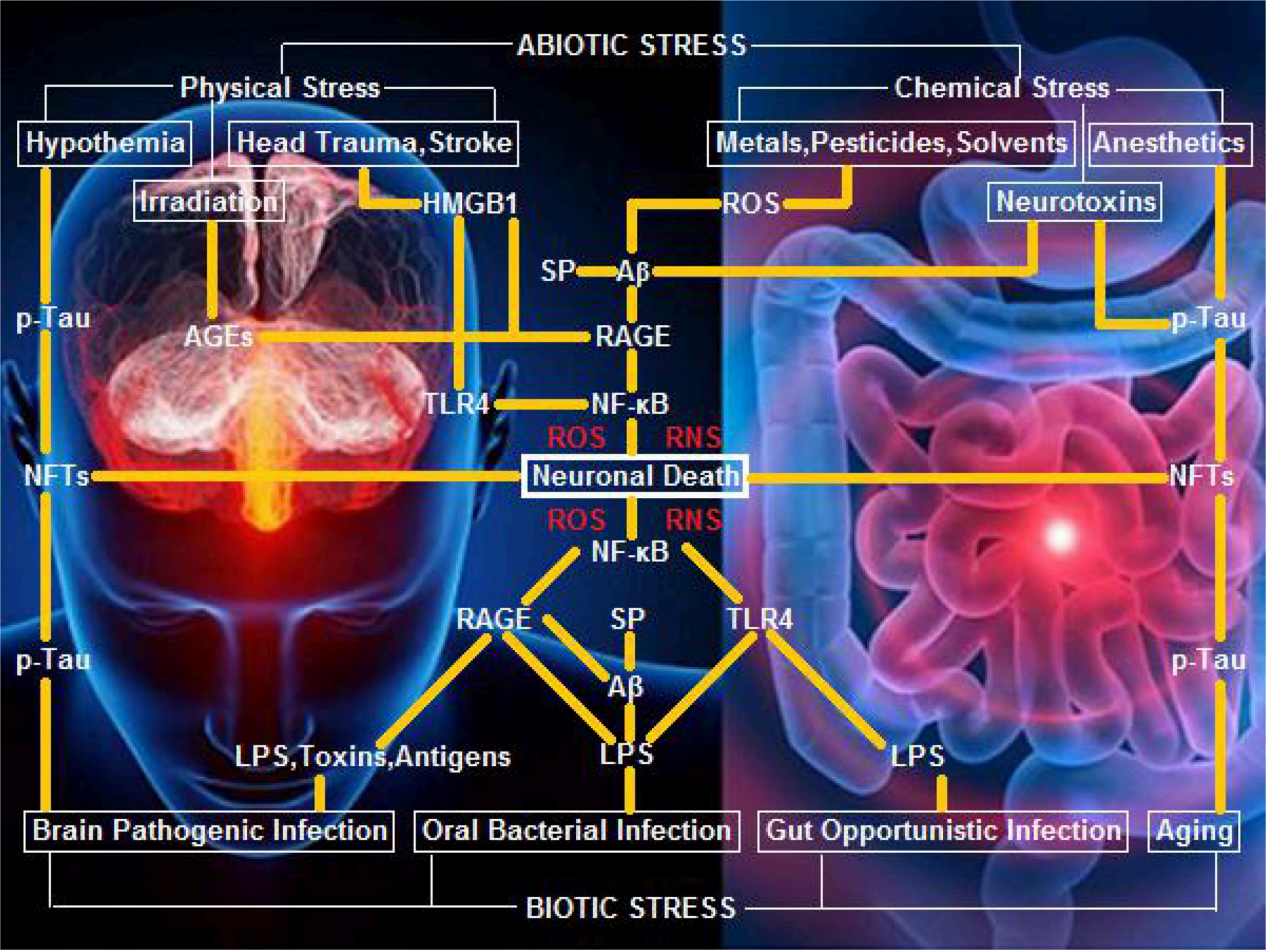
**A hypothetical schematic of biotic/abiotic stress-triggered AD**. Biotic stress from brain, oral, or gut infection can activate NF-κB-primed neuroinflammatory cascades, elicit ROS/RNS burst, and kill neurons and glia via LPS-TLR4/RAGE and Aβ/SP-RAGE interactions and subsequent signaling. Abiotic stress encompassing physical stress (e.g., head trauma, stroke, or irradiation) and chemical stress (e.g., metals, pesticides, solvents, or neurotoxins) can also activate NF-κB-primed neuroinflammatory cascades, elicit ROS/RNS burst, and kill neurons and glia via AGEs-RAGE, HMGB1-RAGE/TLR4, and Aβ/SP-RAGE interactions and downstream signaling. Hypothermia, anesthetics, and aging, can exert a neurotoxic effect upon exposure of neurons and glia to NFTs (the background figure was adopted from the website https://zhidao.baidu.com/daily/view?id=5979).

Our opinion on biotic/abiotic stress-triggered AD links the various stressors to the genesis and progression of AD through a neuroinflammatory signaling cascade, which initiates nuclear factor κB (NF-κB) and induces pro-inflammatory cytokines that evoke potent ROS/RNS burst for neuronal/glial killing. To trigger AD, biotic stressors convey the external biological signals via lipopolysaccharide (LPS)-toll-like receptor 4 (TLR4), LPS-receptor of advanced glycation end products (RAGE), and amyloid β peptide (Aβ)/senile plaques (SP)-RAGE interactions (Yan et al., 1996; Yamamoto et al., 2011). Alternatively, abiotic stressors transduce the external non-biological signals via AGEs-RAGE, high-mobility group protein B1 (HMGB1)-RAGE, and Aβ/SP-RAGE interactions (Mazarati et al., 2011; Horst et al., 2016). Specifically, hypothermia, as well as anesthesia and aging that induce hypothermia, can execute a neurotoxic role to kill neurons and glia via neurofibrillary tangles (NFTs) derived from hyperphosphorylated Tau (p-Tau) (Carrettiero et al., 2015; Figure 1).

Mounting evidence supports that LPS and interferon γ (IFN-γ) activate microglia to induce a pro-inflammatory neurotoxic M1 phenotype, whereas interleukin 4 (IL-4), IL-10, IL-13, and transforming growth factor β (TGF-β) activate microglia to give rise to an anti-inflammatory neuroprotective M2 phenotype (Tang and Le, 2016). Interestingly, we found that electric acupuncture can mimic mechanical wounding to firstly deteriorate LPS-induced AD-like brain pathogenesis, but secondly ameliorate the progressive neurodegeneration in a wounding-healing manner, suggesting a putative conversion from M1 microglia to M2 microglia (He, 2016).

## Biotic stress and AD

Biotic stressors refer to any potential infectious pathogens or opportunistic infectious microbes, including *Chlamydophila pneumoniae* (Balin et al., 1998), *Helicobacter pylori* (Kountouras et al., 2012), *Toxoplasma gondii* (Prandota, 2014), human immunodeficiency virus (HIV; Borjabad and Volsky, 2012), and human cytomegalovirus (HCMV; Lurain et al., 2013). An international team recently urged that cerebral pathogenic infections by herpes simplex virus type 1 (HSV-1), *C. pneumoniae*, spirochetes, and fungi be considered as candidate AD initiators (Itzhaki et al., 2016). Similarly, extracerebral infectious pathogens were also considered as AD triggers; for example, oral pathogenic infections by the periodontal bacteria *Porphyromonas gingivalis* and *Actinomyces naeslundii* were identified as high-risk factors driving development toward AD (Noble et al., 2014; Singhrao et al., 2015). A recent study on gut microbiota dysbiosis indicated that intestinal microbiome alterations are related to the malfunctional motor phenotypes, suggesting the overgrowth of intestinal commensal microbes (i.e., opportunistic infection) acting as a neurodegenerative driver (Scheperjans et al., 2015).

Sulfate-reducing bacteria (SRB), such as the Gram-positive *Firmicutes* and Gram-negative *Proteobacteria*, colonize 50% of human guts (Stewart et al., 2006). Among which *Desulfovibrio piger* was shown as the most common SRB in a surveyed cohort of healthy US adults (Scanlan et al., 2009). Chondroitin sulfate, a daily dietary nutrient available from livestock and poultry products, can increase the abundance of sulfatase-free *D. piger* upon reducing sulfate released from sulfatase-secreting *Bacteroides thetaiotaomicron* (Rey et al., 2013), thereby raising the possibility of *B. thetaiotaomicron* degrading mucin in the gut. Red meat containing heme can also nourish the mucin-degrading bacteria (e.g., *Akkermansia muciniphila*; Ijssennagger et al., 2015). These observations predisposed that gut dysbiosis may lead to the thinned mucosal layers and permeable colon linings, which boost LPS leakage from the gut and entry into the blood stream (Qin et al., 2012).

Factors that link the leaky gut and serum LPS to neurodegenerative diseases include: the plasma level of LPS in patients with neurodegenerative disease is three times higher than in healthy persons (Zhang et al., 2009); and intraperitoneal injections of LPS into mice cause a prolonged elevation hippocampal Aβ levels and lead to cognitive deficits (Kahn et al., 2012). To this end, intranasal LPS infusion was successfully used to establish a neurodegenerative model in rodents (He et al., 2013). According to a recent introduction by Scheperjans (2016) on the relevance of gut microbiota to Aβ deposition, germ-free APP_SWE_/PS1_ΔE9_ mice show mitigated amyloidosis in the brain compared with conventional APP_SWE_/PS1_ΔE9_ mice. While colonization of germ-free APP_SWE_/PS1_ΔE9_ mice with harvested gut microbiota from conventional APP_SWE_/PS1_ΔE9_ mice aggravates cerebral amyloidosis, colonization with gut microbiota from wild-type mice fails to increase cerebral Aβ levels.

Evidence supporting a possible infectious origin of AD is also derived from the sequencing-classified single nucleotide polymorphism (SNP) in apolipoprotein E gene (*APOE*), which is involved in modulating the immune response and infectious susceptibility (Verghese et al., 2011). Genome-wide association studies have revealed that several immune system components including virus receptor genes serve as risk factors for AD (Licastro et al., 2011). For example, cholesterol 25-hydroxylase (CH25H), catalyzing the generation of 25-hydroxycholesterol (25OHC) and inducing the enhancement of innate antiviral immunity, is selectively upregulated by virus infection (Blanc et al., 2013; Liu et al., 2013).

## Abiotic stress and AD

An epidemiological study has associated an increased risk of AD with a medical history of traumatic head injury (Webster et al., 2015). Moreover, brain inflammation seems a common consequence of mechanical insults such as trauma and stroke (Fiebich et al., 2014). Trauma can significantly increase expression of the alarmin HMBG1 (Horst et al., 2016), which in turn activates an inflammatory cascade by stimulating multiple receptors including RAGE and TLR4 (Mazarati et al., 2011). A recent study showed that AD-like model mice, on a diet enriched in AGEs due to irradiation, exhibit significant memory dysfunction, accompanied with the hippocampal deposition of insoluble Aβ42 fragment and AGEs (Lubitz et al., 2016). This latter finding was consistent with the notion that Aβ can activate microglia and induce neurotoxicity by RAGE binding (Yan et al., 1996).

Many naturally occurring and synthesized chemicals such as heavy metals, pesticides, bactericides, and solvents are ROS generators, and therefore are potential initiators of AD (Chin-Chan et al., 2015). A recent study showed that magnetite from air pollution might be an important risk factor for AD; particularly, those magnetite pollutant particles that are <200 nm in diameter can enter the brain directly via the olfactory bulb (Maher et al., 2016). Cyanobacteria or blue-green algae residing in the gut may produce the neurotoxin β-N-methylamino-L-alanine (BMAA), which was implicated in the development of AD (Banack et al., 2010; Brenner, 2013). Chronic dietary exposure to BMAA was identified as a causal factor of neurodegeneration in the Chamorros villagers on the Pacific island of Guam, and vervets (*Chlorocebus sabaeus*) fed with BMAA-dosed fruit were observed to develop neurodegenerative diseases exhibiting Aβ and NFTs (Cox et al., 2016).

It was highlighted that aggregation of p-Tau into NFTs or even development of tauopathies seems an essential consequence of hypothermia as well as anesthetic-induced hypothermia (Planel et al., 2007; Carrettiero et al., 2015). Due to reduced peripheral vasoconstriction, mitigated heat production, and other reasons, the core body temperature of healthy individuals over 60 years of age is 0.4°C lower than adults aged 20–60 years, suggesting that aging should facilitate p-Tau formation by inducing cerebral hypothermia. It was suggested that tau phosphorylation at later stages is mostly a consequence of hypothermia although hyperphosphorylation at early stages may be due to the deregulation of JNK and PP2A (El-Khoury et al., 2016).

## Emerging evidence of Aβ as a responder to infection

In contrast to the conventional assertion of a causative role of Aβ in AD pathogenesis, the peptide was surprisingly recognized as an antimicrobial peptide (AMP) with potent activity against pathogenic infections (Soscia et al., 2010). Aβ has been confirmed to protect mouse, nematode, and cell culture models of AD from fungal and bacterial infections because propagating fibrils mediate the agglutination and eventual entrapment of pathogens. Indeed, bacterial infection by *Salmonella typhimurium* in the brains of transgenic AD mice results in accelerated Aβ deposition, which can co-localize with invading bacteria (Kumar et al., 2016). It was recently reported that a long-term antibiotic treatment regime inducing a prolonged change of gut microbiota decreases Aβ deposition in the APP_SWE_/PS1_ΔE9_ mouse AD model. In the observation, soluble Aβ levels were elevated, plaque-localized glial reactivity attenuated, and microglial morphology altered, suggesting a diversity of gut microbiota regulating host innate immunity, and impacting amyloidosis (Minter et al., 2016).

Aβ was also found to possess antiviral activity against HSV-1 and influenza A (White et al., 2014; Bourgade et al., 2015, 2016). Interestingly, another AMP, β-defensin 1, has similarly shown overproduction in AD patients (Williams et al., 2013). An SNP in human *CH25H* governs both AD susceptibility and Aβ deposition, implying Aβ induction may be a 25OHC target, and also providing a potential mechanistic link between pathogenic infection and Aβ accumulation (Papassotiropoulos et al., 2005; Lathe et al., 2014).

## Aβ as a target for a potential AD remedy

Why Aβ progressively deposits remains largely unknown, but S-nitrosylation of cysteine residues in Aβ-degrading enzymes might be relevant, and nitric oxide (NO) involved. The impact from NO-mediated nitrosative stress was found to prompt the S-nitrosylation of insulin-degrading enzyme (IDE) and dynamin-related protein 1 (Drp1) responsible for Aβ degradation, thus inhibiting Aβ catabolism and hyperactivating mitochondrial fission machinery. The raised Aβ levels and compromised mitochondrial bioenergetics were shown to result in dysfunctional synaptic plasticity and synapse loss in cortical and hippocampal neurons (Akhtar et al., 2016).

Interventions against AD involving eradicating Aβ from brain tissues hold promise in avoiding microglial activation, immune attack, and neuron killing. It was shown that aducanumab, a human monoclonal antibody that selectively targets the aggregated Aβ, enters the brain, binds parenchymal Aβ, and reduces Aβ in a transgenic mouse AD model, and that aducanumab even reduces brain Aβ in patients with prodromal AD after 1 year of monthly intravenous infusions (Sevigny et al., 2016).

Alternatively, prohibition of Aβ formation by impeding the cleavage of APP might also prevent AD. An ongoing human trial is assessing the therapeutic value of the β-secretase inhibitor solanezumab (Sheridan, 2015) although a clinical trial with the γ-secretase inhibitor semagacestat failed just 1 year ago (De Strooper, 2014). The preliminary data indicated that solanezumab can decrease cognitive decline in mild AD by about 30% in a clinical study recruiting 440 subjects (Reardon, 2015).

## Prospectives

Considering Aβ as a pathogenic hallmark of AD, it is anticipated that treatments by monoclonal antibodies to remove Aβ or block APP cleavage would justify optimism and show progress in clinical trials. However, Aβ is unlikely an initiator, and more likely a mediator of AD, so Aβ-targeted interventions should not be an eventual solution to attenuating progressive aggravation toward AD. Once infectious agents have been verified as the primordial etiological cues leading to AD, the more practical medications treating AD should at least include, for example, anti-infection agents such as minocycline (El-Shimy et al., 2015; Budni et al., 2016), anti-inflammation agents such as anhydroexfoliamycin (Leirós et al., 2015) or rapamycin (Siman et al., 2015), and anti-oxidation agents such as allicin (Zhu et al., 2015). With similar importance, modulation of gut microbiota from dysbiosis to homeostasis for the early-phase prophylaxis of AD through personalized diet and prebiotic/probiotic supplementation should also be addressed (Hu et al., 2016).

## Author contributions

QPZ wrote the manuscript. CQL, QZ, and QW critically reviewed the manuscript. All authors read and approved the final version of the manuscript.

### Conflict of interest statement

The authors declare that the research was conducted in the absence of any commercial or financial relationships that could be construed as a potential conflict of interest.

## Abbreviations

Aβ: amyloid-β peptide
AD: Alzheimer's disease
AGEs: advanced glycation end products
AMP: antimicrobial peptide
APOE: apolipoprotein E gene
APP: amyloid precursor protein
CH25H: cholesterol 25-hydroxylase
Drp1: dynamin-related protein 1
HCMV: human cytomegalovirus
HMBG1: high-mobility group protein B1
HIV: human immunodeficiency virus
HSV-1: herpes simplex virus type 1
IDE: insulin-degrading enzyme
IFN: interferon
IL: interleukin
LPS: lipopolysaccharide
NFTs: neurofibrillary tangles
25OHC: 25-hydroxycholesterol
NO: nitric oxide
RNS: reactive nitrogen species
ROS: reactive oxidative species
NF-κB: nuclear factor κB
RAGE: receptor of advanced glycation end products
SP: senile plaques
SRB: sulfate-reducing bacteria
SNP: single-nucleotide polymorphism
TGF: transforming growth factor
TLR4: toll-like receptor 4.
